# Rapid and Effective Vitamin D Supplementation May Present Better Clinical Outcomes in COVID-19 (SARS-CoV-2) Patients by Altering Serum INOS1, IL1B, IFNg, Cathelicidin-LL37, and ICAM1

**DOI:** 10.3390/nu13114047

**Published:** 2021-11-12

**Authors:** Mustafa Sait Gönen, Merve Alaylıoğlu, Emre Durcan, Yusuf Özdemir, Serdar Şahin, Dildar Konukoğlu, Okan Kadir Nohut, Seval Ürkmez, Berna Küçükece, İlker İnanç Balkan, H. Volkan Kara, Şermin Börekçi, Hande Özkaya, Zekayi Kutlubay, Yalım Dikmen, Yılmaz Keskindemirci, Spyridon N. Karras, Cedric Annweiler, Duygu Gezen-Ak, Erdinç Dursun

**Affiliations:** 1 Endocrinology and Metabolism Unit, Department of Internal Medicine, Cerrahpasa Faculty of Medicine, Istanbul University-Cerrahpasa, Istanbul 34098, Turkey; sait.gonen@iuc.edu.tr (M.S.G.); dr.durcan@hotmail.com (E.D.); srdr_shn@hotmail.com (S.Ş.); hmba@iuc.edu.tr (H.Ö.); 2 Brain and Neurodegenerative Disorders Research Laboratories, Department of Medical Biology, Cerrahpasa Faculty of Medicine, Istanbul University-Cerrahpasa, Istanbul 34098, Turkey; merve.alaylioglu@hotmail.com; 3 Department of Infectious Diseases and Clinical Microbiology, Cerrahpasa Faculty of Medicine, Istanbul University-Cerrahpasa, Istanbul 34098, Turkey; ozdemiryusufemre1990@gmail.com (Y.Ö.); ilkerinancbalkan@hotmail.com (İ.İ.B.); 4 Department of Medical Biochemistry, Cerrahpasa Faculty of Medicine, Istanbul University-Cerrahpasa, Istanbul 34098, Turkey; dkonuk@istanbul.edu.tr; 5 Fikert Biyal Biochemistry Laboratory, Cerrahpasa Faculty of Medicine, Istanbul University-Cerrahpasa, Istanbul 34098, Turkey; okannohut@hotmail.com; 6 Department of Anesthesiology and Reanimation, Cerrahpasa Faculty of Medicine, Istanbul University-Cerrahpasa, Istanbul 34098, Turkey; seval.urkmez@istanbul.edu.tr (S.Ü.); ydikmen@iuc.edu.tr (Y.D.); 7 Cerrahpasa Hospital Pharmacy Unit, Cerrahpasa Faculty of Medicine, Istanbul University-Cerrahpasa, Istanbul 34098, Turkey; bernakucukece11@gmail.com; 8 Department of Thoracic Surgery, Cerrahpasa Faculty of Medicine, Istanbul University-Cerrahpasa, Istanbul 34098, Turkey; volkan_kara@yahoo.com; 9 Department of Pulmonary Diseases, Cerrahpasa Faculty of Medicine, Istanbul University-Cerrahpasa, Istanbul 34098, Turkey; borekcisermin@gmail.com; 10 Dermatology and Venerology, Cerrahpasa Faculty of Medicine, Istanbul University-Cerrahpasa, Istanbul 34098, Turkey; zekayikutlubay@hotmail.com; 11 General Directorate of Hospitals, Istanbul University-Cerrahpasa, Istanbul 34098, Turkey; yilmaz.keskindemirci@isuzem.com; 12 Department of Medical Services and Techniques, Health Services Vocational School, Istanbul University-Cerrahpasa, Istanbul 34098, Turkey; 13 National Scholarship Foundation, 55535 Thessaloniki, Greece; 14 Division of Geriatric Medicine, Department of Neuroscience, Angers University Hospital, 49035 Angers, France; CeAnnweiler@chu-angers.fr; 15 Department of Medical Biophysics, Robarts Research Institute, Schulich School of Medicine and Dentistry, The University of Western Ontario, London, ON N6A 3K7, Canada; 16 Department of Neuroscience, Institute of Neurological Sciences, Istanbul University-Cerrahpasa, Istanbul 34098, Turkey

**Keywords:** SARS-CoV-2, COVID-19, vitamin D, cytokine, cathelicidin-LL37, acute respiratory failure

## Abstract

Background: We aimed to establish an acute treatment protocol to increase serum vitamin D, evaluate the effectiveness of vitamin D3 supplementation, and reveal the potential mechanisms in COVID-19. Methods: We retrospectively analyzed the data of 867 COVID-19 cases. Then, a prospective study was conducted, including 23 healthy individuals and 210 cases. A total of 163 cases had vitamin D supplementation, and 95 were followed for 14 days. Clinical outcomes, routine blood biomarkers, serum levels of vitamin D metabolism, and action mechanism-related parameters were evaluated. Results: Our treatment protocol increased the serum 25OHD levels significantly to above 30 ng/mL within two weeks. COVID-19 cases (no comorbidities, no vitamin D treatment, 25OHD <30 ng/mL) had 1.9-fold increased risk of having hospitalization longer than 8 days compared with the cases with comorbidities and vitamin D treatment. Having vitamin D treatment decreased the mortality rate by 2.14 times. The correlation analysis of specific serum biomarkers with 25OHD indicated that the vitamin D action in COVID-19 might involve regulation of INOS1, IL1B, IFNg, cathelicidin-LL37, and ICAM1. Conclusions: Vitamin D treatment shortened hospital stay and decreased mortality in COVID-19 cases, even in the existence of comorbidities. Vitamin D supplementation is effective on various target parameters; therefore, it is essential for COVID-19 treatment.

## 1. Introduction

Since December 2019, the world has been experiencing one of the most striking outbreaks in human history—the COVID-19 pandemic. The main route of COVID-19 transmission was reported as being respiratory droplets and direct contact [1]. It was observed that patients hospitalized in intensive care units (ICU) had high plasma levels of IL-2, IL-7, IL-10, GSCF, IP10, MCP1, MIP1A, and TNFα [2]. Given the natural three-stage clinical course of the disease, inadequate innate immune response in the first stage and immune-mediated damage due to dysregulated immune response in the second stage are considered to be the major determinants of poor outcomes [3]. Several classes of drugs and supplements, including vitamin D, are being evaluated for the treatment of COVID-19, based on the growing evidence regarding the natural history and evolution of the infection obtained from patients [4].

Vitamin D is a secosteroid hormone that has existed on the Earth’s surface for 750 million years and regulates many cellular mechanisms [5,6]. After being produced in the skin by sunlight or dietary intake, it is converted to biologically active 1,25-dihydroxyvitamin D in the liver and kidneys, respectively [7,8]. Although the effects of vitamin D on skeletal and bone metabolism have been well recognized for a long time, its extra-skeletal effects have gradually come into prominence within the last 20 years. In addition, its effects on the regulation of the immune response, oxidative stress, cancer biology, and the nervous system are particularly substantial. [6,9,10,11,12,13].

Vitamin D was used to treat tuberculosis even before anti-mycobacterial drugs were introduced [14]. Numerous cross-sectional studies have been reporting the association between low vitamin D levels and increased rates or severity of various infections, or both, such as influenza [15], bacterial vaginosis [16], and human immunodeficiency virus (HIV) infection [17,18]. The ability of vitamin D to regulate immune response and mitigate the course of acute infections has been highlighted in recent years [11,19,20,21,22].

Vitamin D_3_ replacement is hypothesized to reduce infection-related mortality in intensive care units (ICUs) via increasing hemoglobin concentrations, reducing serum hepcidin concentrations, improving oxygenation on the cellular level, and reversing lung damage [23,24,25,26,27,28,29]. Recently, studies have demonstrated an association between vitamin D deficiency and the severity and increased mortality of COVID-19. Vitamin D deficiency has been associated with more severe clinical forms of COVID-19 [30,31,32,33]. A study reported that patients supplemented with 10,000 IU/daily vitamin D in COVID-19 presented fewer symptoms than non-supplemented patients [34].

In this study, we aimed to: (1) investigate whether vitamin D deficiency is a risk factor in the clinical course of COVID-19 infection; (2) establish an acute (bolus) treatment protocol to increase serum vitamin D (25 hydroxy-vitamin D-25OHD) to sufficient levels (>30 ng/mL); (3) evaluate the effectiveness of vitamin D_3_ supplementation in the COVID-19 treatment, and develop a recommendation for routine treatment of patients in varying clinical severities; (4) reveal the novel potential mechanisms that vitamin D acts on modulating COVID-19 immune response and augment treatment success.

## 2. Materials and Methods

### 2.1. Study Design and Patient Groups

**Figure 1:**
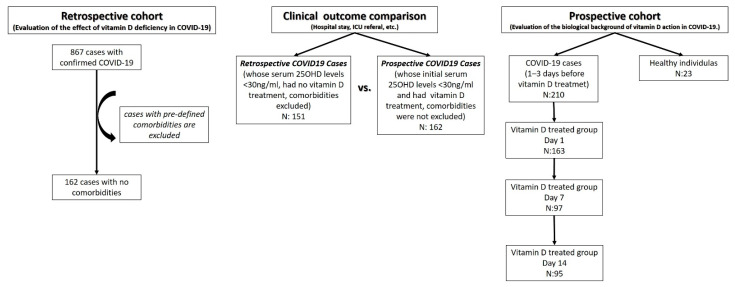
The study design and patient groups.

The study was conducted in two stages. The flow chart of patient recruitment is shown in Figure 1, in a consort diagram. In the retrospective part, data of 867 patients admitted to Istanbul University-Cerrahpasa (Cerrahpasa Faculty of Medicine) Faculty Hospital between 7 March and 22 May 2020, with a confirmed diagnosis of COVID-19, based on clinical and PCR findings, were analyzed. Considering that other diseases may affect the vitamin D status, severity, or progression of COVID-19 infection, cases with comorbidities such as cancer, thyroid or kidney disease, or cardiovascular or autoimmune diseases were excluded. This left 162 cases in the first part of the study (Figure 1). All patients received anti-virals (hydroxychloroquine, azithromycin, oseltamivir, and favipiravir) and some received anti-cytokine (tocilizumab) treatment, in case of indication, according to current national guidelines. The first stage of the study was conducted to evaluate the effect of serum vitamin D (25OHD) on status in COVID-19.

The second part, which was designed as a prospective randomized controlled study, involved 210 individuals diagnosed with COVID-19 and 23 healthy individuals (mean age 35.5 ± 8.2; range 26–48; 65.2% female). A total of 163 COVID-19 cases whose serum 25OHD levels were less than 30 ng/mL received vitamin D3 (cholecalciferol) treatment, according to the protocol (Table 1), which was created by compiling evidence-based data from the literature [23,24,25,26], while 47 cases had no vitamin D treatment at all. A total of 95 out of 163 cases who had vitamin D supplementation were followed for at least 14 days. We should note that the patients that were treated with vitamin D were vitamin D deficient or insufficient (serum 25OHD levels < 30 ng/mL). The safety of the treatment was checked by monitoring serum 25OHD and Ca^2+^ levels (for toxicity and calcification) weekly. In this second part, peripheral blood samples were collected from all patients 1–3 days before treatment and from patients who received vitamin D treatment on day 7 (D7) and day 14 (D14) of the treatment (Figure 1). The second stage of the study was conducted to evaluate the biological background of the effect of vitamin D treatment in COVID-19.

Clinical outcomes, such as hospital stays and ICU referrals, were evaluated in a retrospective cohort to assess the effect of serum vitamin D status, and in both retrospective and prospective cases to evaluate the effect of vitamin D treatment (Figure 1).

Participants in the present study were treated according to the current national COVID-19 guidelines, which did not have any recommendation regarding vitamin D supplementation at the time of study or during the manuscript writing process. The study adhered to the ethical principles for medical research involving human participants, described in the World Medical Association’s Declaration of Helsinki. The study was approved by the Ethics Committee of Istanbul University, Cerrahpasa, and Republic of Turkey Ministry of Health (Approval Number: Mustafa Sait Gönen-2020-05-06T19_51_05). Signed informed consent was obtained from all study participants.

### 2.2. Target Parameters

The relation between vitamin D supplementation and disease parameters, such as gender, age, hospitalization time, ICU (intensive care unit) stay, CBC (Complete blood count), Urea, Creatinine, Sodium, Potassium, Chlorine, AST, ALT, Total Bilirubin, LDH, CPK, D-dimer, Ferritin, troponin, and CRP were noted in hospital records and gathered electronically. The analysis was based on comparing these between 2 groups. The data for the aforementioned parameters was gathered from the database of Hospitals General Directorate of Cerrahpasa Faculty of Medicine.

The molecular infrastructure of vitamin D’s effectiveness in the COVID-19 treatment protocol was investigated with vitamin D metabolism (25OHD, vitamin D binding protein-DBP, parathormone-PTH, and Ca^2+^), immune response (cathelicidin-LL-37, Interleukin-IL1b, IL6, IL17, Interferon gamma-INFg, and calcium binding protein B-S100B), and endothelial function (Intercellular Adhesion Molecule 1-ICAM1, Vascular cell adhesion protein 1-VCAM1, nitric oxide-NO, and Nitric Oxide Synthase 1-NOS1)-related parameters. DBP, cathelicidin LL-37, IL1b, IL6, IL17, INFg, S100B, ICAM, VCAM, NO, and NOS parameters were investigated by ELISA, 25OHD, PTH, and Ca^2+^ with CLIA methods. The kits that were used were the following: Elecsys Vitamin D total II (7464215190, Roche, detection range: 3–100 ng/mL, sample dilution factor (SDF): 2); Elecsys PTH (11972103122, Roche, detection range: 1.20–5000 pg/mL, sensitivity: 6.0 pg/mL, SDF: 1); Calcium Gen.2 (05061482190, Roche, detection range: 0.20–5.0 mmol/L); Human LL-37 (Antibacterial Protein LL-37) ELISA Kit (E-EL-H2438, Elabscience, detection range: 1.56–100 ng/mL, sensitivity: 0.94 ng/mL, sample dilution factor (SDF): 1); IL-1 beta Human ELISA Kit (BMS224-2, Thermo, detection range: 3.9–250 pg/mL, sensitivity: 0.3 pg/mL, SDF: 2); Human IL-6 ELISA Kit (BMS213-2, Thermo, detection range: 1.56–100 pg/mL, sensitivity: 0.92 pg/mL, SDF: 2); Human IL-17(Interleukin 17) ELISA Kit (E-EL-H0105, Elabscience, detection range: 31.25–2000 pg/mL, sensitivity: 18.75 pg/mL, SDF: 1); Human IFN-gamma ELISA Kit (BMS228, Thermo, detection range: 1.6–100 pg/mL, sensitivity: 0.99 pg/mL, SDF: 2); Human S100B(S100 Calcium Binding Protein B) ELISA Kit (E-EL-H1297, Elabscience, detection range: 31.25–2000 pg/mL, sensitivity: 18.75 pg/mL, SDF: 1); Human ICAM-1(intercellular adhesion molecule 1) ELISA Kit (E-EL-H6114, Elabscience, detection range: 0.31–20 ng/mL, sensitivity: 0.19 ng/mL, SDF: 1); Human VCAM-1/CD106 (Vascuolar Cell Adhesion Molecule 1) ELISA Kit (E-EL-H5587, Elabscience, detection range: 1.56–100 ng/mL, sensitivity: 0.94 ng/mL, SDF: 1); nitrate–nitrite (index of total NO production) Colorimetric Assay Kit (780001, Cayman, detection limit: 2.5 µM, SDF: 2); Human NOS1/nNOS (Nitric Oxide Synthase 1, Neuronal) ELISA Kit (E-EL-H0742, Elabscience, detection range: 0.16–10 ng/mL, sensitivity: 0.10 ng/mL, SDF: 1); Human DBP (Vitamin D Binding Protein) ELISA Kit (E-EL-H1604, Elabscience, detection range: 3.91–250 ng/mL, sensitivity: 2.35 ng/mL, SDF: 1).

### 2.3. Statistics

We used the SPSS 24 or GraphPad Prism 7.0a (GraphPad Software, Inc. San Diego, CA, USA) program for the biostatistical analysis of this study. For pairwise comparison, the data were compared using the independent sample t-test when the data were normally distributed and the Mann–Whitney U test when the data were not normally distributed. *p* < 0.05 was accepted as statistically significant. In comparisons of more than two groups, whether the data is normally distributed and whether the difference between the obtained standard deviations is significant were determined firstly by one-way ANOVA, followed by Tukey–Kramer multiple comparison tests, or, for multiple comparisons, Kruskal Wallis then Dunn’s multiple comparison tests were used. *p* < 0.05 was accepted as statistically significant. The effect of age or gender difference on categorized data was adjusted with binary logistic regression analysis. When required, the corrected effect size was calculated with Glass’ delta (GΔ), where 0.2 is suggested as a small effect size, 0.5 as medium, and 0.8 is a larger effect [35,36]. The overall corrected effect size for multiple comparisons was calculated as the average of individual GΔs determined for each significant outcome [36]. In the prospective study, age and sex adjustment was performed with one way analysis of covariance (ANCOVA) and the observed power was stated. Pearson correlation was used in normally distributed groups, and Spearman correlation was used in non-normally distributed groups, for the correlation analysis between parameters.

## 3. Results

### 3.1. The Effect of Serum Vitamin D Status on Clinical Outcomes of Retrospective Cases

The rate of ICU admission was 17.53% (152 out of 867) in the whole cohort and 4.94% (8 out of 162) in the sub-group had no comorbidities. Co-existing diseases increased the risk of ICU admission by 3.6 times (*p* = 0.0007, 95%CI: 1.7100 to 7.3705, OR: 3.55, post-hoc power: 99.9%). The rate of ICU admission was not significantly different in cases with serum 25OHD levels either lower or higher than 12 ng/mL (*p* = 0.502), regardless of comorbidity (Table 2). ICU admission was not significantly different between COVID-19 cases with no comorbidities and COVID-19 cases with no comorbidities but having serum 25OHD levels higher than 12 ng/mL (*p* = 0.7459, 95% CI: 0.3228 to 4.8481, OR: 1.25).

Mean ICU stay in COVID-19 cases, including those with co-existing diseases, was 7.47 ± 7.35, N:152. Mean ICU stay in COVID-19 cases excluding those with co-existing diseases while having serum 25OHD levels lower than 12 ng/mL, was 17.80 ± 6.91, N:5. The ICU stay duration of this group was significantly higher than that of COVID-19 cases including co-existing diseases (*p* = 0.0042, 95% CI: 3.736 to 16.916, post hoc power: 90.7%, Glass’ Δ: 1.41). Given the number of COVID-19 cases, excluding those with co-existing diseases whose serum 25OHD levels were higher than 12 ng/mL and who went into ICU, were less than five, we were not able to analyze the ICU stay in this group.

The rate of mortality was 11.19% (97 out of 867) in the whole cohort, including patients with comorbidities. The mortality rate of prospective cases who also had comorbidities but received vitamin D treatment was 5.5% (9 out of 162). Having vitamin D treatment decreased the mortality rate 2.14 times (*p* = 0.03, 95%CI: 1.0585 to 4.3327, OR: 2.14, post-hoc power: 61.0%).

### 3.2. Retrospective Study

The study samples were investigated in 4 groups: the cases with serum 25OHD levels <12 ng/mL (L1), 12–20 ng/mL (L2), 20–30 ng/mL (L3), or >30 ng/mL (L4), first. The results indicated that, besides serum 25OHD levels, the parameters that were significantly different between groups were serum Ca^2+^ and nitrate–nitrite (Table 2). When study samples were dichotomized according to serum 25OHD levels, we created two groups—the cases with serum 25OHD levels <12 ng/mL and >12 ng/mL—in order to increase the power of the study. We observed that serum DBP and NOS1 levels were significantly high and PTH levels was significantly low in cases whose serum 25OHD levels were >12 ng/mL. The differences between the two groups were the nearly significant Ca^2+^ and creatinine levels (Table 3).

### 3.3. The Effect of Vitamin D Treatment on Clinical Outcomes: Untreated Retrospective Cases vs. Vitamin D Treated Prospective Cases

Descriptive analyses of age, sex, hospitalization (stay) period, and admission to ICU in COVID-19 cases that had or did not have vitamin D treatment are shown in Table 4. The cases that stayed in hospital longer than 8 days were significantly less in COVID-19 cases that had vitamin D treatment compared with the ones that had no vitamin D treatment (*p* = 0.02) (Table 4); however, the retrospective cohort and prospective cohort differed by means of age gender distribution (*p* = 0.004, *p* = 0.008; respectively), given that the data adjusted for age and sex. The binary logistic regression analysis indicated that the significance of hospital stay (< or >8 days) did not depend on gender. Retrospective COVID-19 cases (without additional disease, without vitamin D treatment, and serum 25OHD <30 ng/mL) had the 1.9-fold increased risk of hospitalization longer than 8 days (*p* = 0.007, OR: 1.91, 95%CI: 1.19–3.06). Increased age was also a risk factor for hospitalization longer than 8 days (*p* = 0.023, OR: 1.03, 95%CI: 1.00–1.06) (Table 4).

### 3.4. Prospective Study (the Biological Background of Vitamin D Treatment)

#### 3.4.1. Vitamin D Treatment Formula

After following the treatment protocols (Table 1) given in this article, the increase in a patient serum 25OHD levels within 14 days might be predicted with the formula “y = 8.63 ln(x) + 13.66”, where x = the initial level of serum 25OHD and y = the predicted serum 25OHD levels 14 days after treatment. The formula was extracted from the graphics of the COVID-19 cases that include the serum 25OHD levels in days 1, 7, and 14 of the treatment protocol. The predicted values of serum 25OHD (*n*: 142, 34.59 ± 5.27) indicated no significant difference for the comparison with the D14 measured serum 25OHD levels (*n*: 95, 35.46 ± 10.92), (*p* > 0.05, 95%CI: −1.521 to 3.251). The serum 25OHD levels of COVID-19 cases (day 14 of vitamin D treatment—D14) was significantly higher than that of COVID-19 cases (1–3 days before vitamin D treatment -C), (*p* < 0.001, Table 5).

#### 3.4.2. Mean Comparisons

The serum 25OHD levels of healthy individuals were higher than those in COVID-19 cases that did not receive vitamin D treatment and those who received vitamin D treatment for 14 days. On the other hand, the serum 25 OHD levels of the COVID-19 cases on the 7th and 14th days were higher than the COVID-19 cases 1–3 days before the treatment, which did not receive vitamin D treatment. The Ca^2+^ level of cases was relatively increased on the 14th day after treatment, yet it was statistically significant. Given that the fact that the mean value of serum 25OHD levels begin with 16.62 ± 11.85 and only reached 35.46 ± 10.93, which is far below the possible toxic dose of 100 ng/mL within two weeks, and the serum Ca2+ levels did not increase significantly on the 14th day, the treatment protocol was accepted as safe. Considering the PTH level, it was observed that, although the PTH levels of COVID-19 cases that did not receive vitamin D supplementation were relatively high, this level came close to healthy individuals in COVID-19 cases on the 14th day of vitamin D supplementation. It was determined that serum nitrate–nitrite levels were higher in COVID-19 cases on the 7th and 14th day of the treatment, compared with controls. A similar situation was observed for NOS1 as well. While the DBP level was higher in the cases that did not receive supplementation, compared with the controls, it was observed that the cases that received the supplement gradually decreased and regressed to the control levels on the 7th and 14th days. IL1B level was higher in all case groups compared with controls. Although this was not statistically significant, the IL6 level on the 14th day was found to be lower than the cases that did not take vitamin D supplements. IFNg level remained high in all cases compared with controls. IL17 level was lower in all cases compared with controls. Although the LL37 level remained high in all case groups compared with controls, it was significantly reduced on the 7th and 14th days of supplementation compared with the non-supplemented subjects. S100B level was found to be high in cases that did not take vitamin D supplements compared with controls. It was observed that ICAM1 levels were higher in COVID-19 cases on the 7th and 14th day of the treatment compared with controls. Moreover, cases on the 14th days of the treatment had higher ICAM1 levels than cases that did not receive supplementation.

The routine blood parameters were analyzed only in cases of COVID-19 that did not take vitamin D supplements and did take supplements, given they were not followed in healthy subjects. It was observed that the ALT level remained higher on the 7th and 14th days compared with those who did not take supplements. No such change was observed for AST. While the CRP level was high in the cases who did not take the supplement and, in the cases on the 1st day of the supplementation, it was observed that it decreased significantly in the cases on the 7th and 14th days. No change in serum creatinine levels was observed. It was observed that the sodium level remained high on the 7th and 14th days. There was no significant difference between the case groups regarding urea, ferritin, hemoglobin, and D-dimer levels. However, it was observed that the leukocyte and platelet levels were high on the 14th day of the cases that received vitamin D supplements, while the fibrinogen level was significantly lower. Detailed statistical analyses with numbers are mentioned in Table 6.

#### 3.4.3. Correlation Analysis

While a positive correlation was observed between serum 25OHD level and serum Ca^2+^ level in COVID-19 cases that did not receive vitamin D supplementation, no such correlation was observed in healthy controls and cases on the 7th and 14th days of supplementation. While a negative correlation was observed between serum 25OD level and serum PTH level in healthy controls, in cases that did not receive supplementation, and on the 7th day of supplementation, it was observed that this correlation disappeared on the 14th day of supplementation. A negative correlation was observed between serum 25OD level and serum nitrate–nitrite levels, only in cases that did not receive supplementation. When NOS1 was examined, it was observed that serum 25OHD level and NOS1 level were not correlated in healthy controls but negatively correlated in cases that did not receive supplementation and positively correlated in cases that received supplementation. While DBP was not correlated with 25OHD in healthy subjects, it was found to be positively correlated in all case groups. While serum 25OHD level and serum IL1B level were not correlated with the control group in the cases who received supplementation, it was observed that they were positively correlated in the cases who did not receive the supplement. No correlation was detected between IL6 and serum 25OHD levels in any group. While serum 25OHD level and serum IFNg level were not correlated in the control group or the cases receiving supplementation, it was negatively correlated in those who did not receive the supplement. No correlation was detected between IL17, S100B, VCAM1, and serum 25OHD levels in any group. While serum 25OHD level and serum LL37 level were not correlated in the control group or in cases that did not receive supplementation, they were positively correlated in vitamin D supplemented cases. While serum 25OHD level and serum ICAM1 level were not correlated in the control group or in the cases who received supplementation, they were negatively correlated in those who did not receive the supplement. Detailed statistical analyses with numbers are mentioned in Table 7.

## 4. Discussion

The present study aimed to evaluate the effectiveness of vitamin D3 supplementation in COVID-19 treatment and reveal the potential mechanisms of vitamin D on COVID-19. Our results indicated that vitamin D treatment shortened the hospitalization period, decreased the mortality rate, and that the effect of vitamin D in COVID-19 might involve regulation of INOS1, IL1B, IFNg, cathelicidin-LL37, and ICAM1.

### 4.1. The Efficiency of Vitamin D Supplementation

Although vitamin D supplementation is a well-established subject in bone health and bone-related diseases, the knowledge on its effects on extra-skeletal functions is not well established. When vitamin D deficiency was reported to increase the risk of COVID-19 disease [30], we established a vitamin D supplementation protocol from the existing literature, that focused on lung damage, reduced oxygen saturation, and sepsis [23,24,25,26]. Our treatment protocol increased the serum 25OHD levels significantly to above 30 ng/mL within two weeks. The Ca^2+^ level of cases was relatively increased on the 14th day after treatment, yet it was statistically significant after age and sex adjustment. PTH levels of COVID-19 cases who did not receive vitamin D supplementation were relatively high; moreover, this level came close to healthy individuals in COVID-19 cases on the 14th day of vitamin D supplementation. DBP level was higher in the cases that did not receive supplementation compared with the controls. However, the cases that received the supplement gradually decreased and regressed to the control levels on the 7th and 14th days. Therefore, we may conclude that the treatment protocol was safe, efficient, and functioning effectively. This protocol might be presented as a way of safe, fast, and significant elevation of serum vitamin D levels in adults in 14 days.

### 4.2. Vitamin D, Iron, and Hemoglobin

The relationship between iron and vitamin D has been evaluated in three studies [23,24,25,26]. Two studies found a significant positive correlation between serum iron and basal vitamin D concentration, hematocrit, and transferrin saturation [24,26]. In another study, low hemoglobin (Hb) and transferrin saturation was observed in babies with low 25(OH)D and low 24.25(OH)2D [25]. On the other hand, anemia is quite common in critical illnesses. Approximately two-thirds of ICU adolescent patients develop anemia in the first week of admission and anemia at admission to ICU [37,38]. Anemia is associated with an increased low oxygen-carrying capacity and cardiovascular morbidity, potentially prolonging mechanical ventilation duration, thus increasing the total risk for mortality [27]. A study of 475 patients hospitalized in intensive care units showed that, in patients with severe vitamin D deficiency (<12 ng/mL), an oral or nasogastric-mediated single dose of 540,000 IU vitamin D3 administration significantly decreased mortality compared with the placebo group. This effect was not observed in those with low vitamin D levels (20–13 ng/mL) [39]. In another study, it was shown that in adults hospitalized in ICU, 100,000 IU daily for five days and a total of 500,000 IU vitamin D3 treatment increased hemoglobin concentrations over time and acutely decreased serum hepcidin concentrations. This effect was not observed in patients receiving 50,000 IU per day, totaling 250,000 IU [40]. 

Either retrospective or prospective part of our study, there was no significant difference between case groups regarding urea, ferritin, hemoglobin, and D-dimer levels.

### 4.3. Vitamin D and Sepsis

Sepsis is a life-threatening organ dysfunction caused by the host in response to infection and is still the leading cause of death in critically ill patients [28]. In recent years, studies have shown that vitamin D deficiency or insufficiency is common in critically ill patients, particularly in severe sepsis cases [29]. It is thought that the relationship between vitamin D and sepsis can be explained by mechanisms that work through regulation of the immune system and inflammation, endothelial cell protection, and carbon monoxide regulation [28].

Results from a meta-analysis examining twenty-four studies showed that cases of sepsis had significantly lower vitamin D levels in all populations, especially in Caucasians and Africans, compared with cases without sepsis. Vitamin D levels in sepsis cases were not associated with ALB, PLT, WBC, mortality, PCT, BMI, male to female ratio, IL-6, and CRP levels, nor were they associated with death due to sepsis. However, the meta-analysis suggests that vitamin D deficiency may be a biomarker of sepsis risk in all populations, independent of other variables [29]. Vitamin D administration has been shown to reverse lung injury and reduce the decrease in oxygen saturation in animals with an intratracheal lipopolysaccharide (IT-LPS) sepsis model [29].

In our study, while the CRP level was high in the cases that did not receive vitamin D treatment and in the cases on the 1st day of the treatment, it decreased significantly in the cases on the 7th and 14th days. However, the leukocyte and platelet levels were high on the 14th day of the cases that received vitamin D treatment, whereas the fibrinogen level was significantly lower. It was observed that the ALT level remained higher on the 7th and 14th days compared with those who did not take supplements. No such change was observed for AST.

### 4.4. Vitamin D and COVID-19

In a study conducted on 212 COVID-19 cases, the probability of having a mild disease is correlated to high levels of vitamin D. On the contrary, as the vitamin D levels decrease, the risk of severe disease increases [30]. Another study demonstrates an association between vitamin D deficiency and severity and increased mortality of COVID-19 [31]. A study reported that supplementation of 10,000 IU/daily vitamin D in COVID-19 patients presented fewer symptoms compared with those non-supplemented on the 7th and 14th day of follow-up, and 10,000 IU/daily vitamin D supplementation for 14 days was sufficient to increase vitamin D serum concentrations in a western Mexican population [34]. A retrospective study done in the United Arab Emirates showed that vitamin D levels lower than 12 ng/mL were significantly associated with a higher risk of COVID-19 severity and of death [32]. A systematic review and meta-analysis study indicated a link between serum vitamin D levels and COVID-19 severity and mortality [33]. In our study, ICU referral did not significantly differ between COVID-19 cases without any comorbidities and COVID-19 cases with no other comorbidities but having serum 25OHD levels higher than 12 ng/mL. Besides, there was no significant difference between cases with serum 25OHD levels >12 ng/mL and those with 25OHD levels of <12 ng/mL in ICU stay. COVID-19 cases with no comorbidities, who had no vitamin D treatment, and whose serum 25OHD level was <30 ng/mL had the 1.9-fold increased risk of having hospitalization longer than 8 days compared with the COVID-19 cases with comorbidities, whose serum 25OHD level was <30 ng/mL, who had vitamin D treatment. At this point, it is important to note that vitamin D treatment shortened hospital stay even for the COVID-19 cases in our treatment group that had comorbidities. Besides, having vitamin D treatment decreased the mortality rate 2.14 times, even in the presence of comorbidities.

A recent study suggested impaired vitamin D metabolism and elevated PTH levels eight weeks after onset. The study indicated no association between low vitamin D levels and persistent symptom burden, lung function impairment, ongoing inflammation, or more severe CT abnormalities. They suggested that vitamin D deficiency is frequent among COVID-19 patients but not associated with disease outcomes. Cases with severe disease displayed a disturbed parathyroid–vitamin D axis within their recovery phase. [41]. In a study by Mazziotti et al., it was shown that vitamin D deficiency with secondary hyperparathyroidism was associated with acute hypoxemic respiratory failure in COVID19 patients [42]. In our study, PTH levels of COVID-19 cases who did not receive vitamin D supplementation were relatively high. Yet, this level came close to healthy individuals in COVID-19 cases on the 14th day of vitamin D supplementation. 

A recent study reported that serum calcium and vitamin D levels in COVID-19 patients were lower than in healthy individuals [43]. Osman et al. showed that hypocalcemic COVID-19 patients had longer hospitalization duration and higher severity of the disease, yet they could not find a link between vitamin D status and COVID-19 [44]. Our results showed that the Ca^2+^ level of cases was relatively increased on the 7th and 14th day after treatment, yet it was not statistically significant

It is known that vitamin D acts as a regulator of many cytokines in many cell types of the immune system and in many diseases [11,19,20,21]. Vitamin D enhances innate cellular immunity in part by stimulating many antimicrobial peptides, including human cathelicidin, LL-37, and defensins [45]. In our study, the serum cathelicidin-LL37 level was higher in all case groups compared with controls but was significantly decreased on day 7 and 14 of supplementation compared with non-supplemented cases. Although vitamin D was named as a vitamin, it is rather a secosteroid hormone [10]. Vitamin D can exhibit both anti-inflammatory and pro-inflammatory responses simultaneously, depending on cell, tissue, or microenvironment. This might be a regulatory response of vitamin D to attenuate LL-37 up-regulation in COVID-19 patients.

Vitamin D also regulates the cellular immune response by reducing the cytokine storm stimulated by the innate immune response. As seen in COVID-19, the innate immune response stimulates the release of both pro-inflammatory and anti-inflammatory cytokines in response to viral and bacterial infections [2]. Vitamin D levels are associated with cytokines such as IL-1, IL-6, IL-10, and TNFα [5]; additionally, vitamin D can reduce pro-inflammatory TH1 cytokines such as TNFα and IFNg, and increase anti-inflammatory cytokines released from macrophages [45,46,47]. In this respect, it is known that it can also regulate the adaptive immune response [14].

IL17 and IL8 are accepted as significant contributors in the pulmonary inflammatory reaction to infectious agents that induce a Th1/Th17 response. These cytokines increase vascular permeability and allow the intense neutrophilic infiltrates to give a response to viral infection. A study indicated the G allele of rs3819025 correlated with higher tissue expression of IL-17A in the COVID-19 cases [46]. In our study, serum IL17 levels of all COVID-19 cases, whether they received vitamin D supplementation or not, remained low compared with controls. A retrospective study investigating cytokine gene expression in COVID-19 patients showed that IL1 β mRNA expression levels were increased in COVID-19 patients compared with healthy individuals [47]. Our results indicated that the IL1β level remained higher in all COVID-19 case groups compared with controls. Although not statistically significant, we observed that the IL6 level on the 14th day was below that of the cases that did not take vitamin D supplements. A systematic review and meta-analysis study reported that elevated IL6 levels are associated with COVID-19 severity [48]. In the study of Li et al., COVID-19 patients had higher IL6 mRNA expression levels compared with healthy individuals [47]. Lakkireddy et al. found that COVID-19 patients with hypovitaminosis D had evaluated IL6 levels and IL6 levels were reduced in patients supplemented with 60,000 IUs/daily of vitamin D for 8–10 days compared with the patients who received standard treatment [49].

IFNg serum levels were found to be decreased in COVID-19 compared with both macrophage activation syndrome (MAS) and secondary hemophagocytic lymphohistiocytosis (sHLH), in which cytokine storm is seen [50]. In a study that investigated the expression levels of several cytokine genes in leukocytes of ICU and non-ICU COVID-19 patients, it was shown that IFNg had higher expression levels in non-ICU than in ICU patients [51]. Our data showed IFNg levels were higher than expected in all groups, regardless of their vitamin D supplement status.

NOS1 and S100B were selected as neuronal markers for COVID19 cases. Nitric oxide (NO) functions as an immune mediator and plays an important role in vascular and inflammatory lung diseases [52]. Although a relation was not investigated with neuronal nitric oxide synthase (NOS1), vitamin D was suggested to be the regulator of inducible nitric oxide synthase (NOS2) [53,54] and endothelial nitric oxide synthase (NOS3) [55]. The final products of NO are nitrite and nitrate. The best index of total NO production is accepted as the sum of both nitrite and nitrate (nitrate–nitrite). In our study group, we determined that serum nitrate–nitrite levels, the metabolites of NO, and NOS1 levels were higher in all COVID-19 cases compared with controls. However, the serum 25OHD level and NOS1 level were not correlated in healthy controls but negatively correlated in cases that did not receive supplementation, and positively correlated in cases that received supplementation in our study. Higher serum nitrate levels were also reported in non-surviving COVID-19 patients compared with surviving patients [56]. S100B is a Ca^+2^ binding protein mainly expressed by astrocytes and is used to detect glial activation or death in neurological disorders, or both [57]. Elevated serum levels of the S100B protein were found in COVID-19 patients, reflecting an increased blood–brain barrier permeability [58]. Serum S100B levels were found to be associated with COVID-19 severity [59]. In our study, we found that S100B levels were higher in all COVID-19 cases compared with controls.

In our study, it was observed that ICAM1 levels were higher in COVID-19 cases on the 7th and 14th days of the treatment compared with controls. Moreover, cases on the 14th days of the treatment had higher ICAM1 levels than cases who did not receive supplementation. Although VCAM1 levels were gradually increased in all COVID-19 cases compared with controls, it was not statistically significant. Serum levels of VCAM-1 were found to be higher in COVID-19 patients than in non-COVID-19 patients [60]. Li et al. showed that serum VCAM-1 and ICAM-1 levels were elevated in mild and severe COVID-19 cases compared with healthy subjects [61]. Kessel et al. were found that serum levels of ICAM-1 were increased in COVID-19 patients compared with both (MAS) and (sHLH) patients [50]. In COVID-19-related acute respiratory distress syndrome, plasma ICAM-1 levels were found to be higher in non-survivors than in survivors [62]. 

The response of vitamin D in individuals with already high vitamin D levels may be more effective than the response of vitamin D, which is increased in a short time with treatment. However, in individuals whose vitamin D level is moved to the normal range by treatment, a longer time may be required to observe the effect of this level on cytokines. This reveals the importance of having normal vitamin D levels for a healthy life.

## 5. Conclusions

In conclusion, it has been determined that comorbidity is the most important factor in the duration of admission to intensive care unit and hospital stay in the course of COVID-19. It was observed that the length of stay in the ICU was significantly higher in COVID-19 cases without comorbidities, with serum 25OHD levels lower than 12 ng/mL, than in COVID-19 cases with comorbidities. Vitamin D treatment shortened hospital stay in COVID-19 cases even in the existence of comorbidities. Having vitamin D treatment decreased the mortality rate by 2.14 times. It has been determined that vitamin D supplementation is effective on various targeted parameters; therefore, it is an important parameter for the course of COVID-19, and serum vitamin D levels and correlation analyses between these parameters confirm this inference. However, considering the parameters and the chronic characteristics of the disease, it became necessary to examine the long-term effects of vitamin D supplementation on the long-term effects of COVID-19, including full recovery duration and irreversible organ damage. Moreover, it is important to note that further investigations with a high number of healthy individuals and more detailed patient data might widen knowledge on the potential effects of vitamin D.

## Figures and Tables

**Table 1:** Vitamin D3 (cholecalciferol) treatment protocol.

COVID-19 VITAMIN D (CHOLECALCIFEROL) SUPPLEMENTATION										
Patient Definition		DAY 1	DAY 2	DAY 3	DAY 4	DAY 5	DAY 6	DAY 7	TOTAL PERIOD	TOTAL DOSE
INPATIENT	Serum 25OHD level < 12 ng/mL	100.000 IU	10.000 IU	10.000 IU	10.000 IU	10.000 IU	10.000 IU	10.000 IU	14 Days	320.000 IU
	Serum 25OHD level 20–12 ng/mL	100.000 IU	5.000 IU	5.000 IU	5.000 IU	5.000 IU	5.000 IU	5.000 IU	14 Days	260.000 IU
	Serum 25OHD level 20–30 ng/mL	100.000 IU	2.000 IU	2.000 IU	2.000 IU	2.000 IU	2.000 IU	2.000 IU	14 Days	224.000 IU
ICU PATIENT	Serum 25OHD level < 12 ng/mL	100.000 IU	100.000 IU	100.000 IU	100.000 IU	100.000 IU			5 Days	500.000 IU
	Serum 25OHD level 20–12 ng/mL	100.000 IU	100.000 IU	100.000 IU	100.000 IU				4 Days	400.000 IU
	Serum 25OHD level 20–30 ng/mL	100.000 IU	100.000 IU	50.000 IU					3 Days	250.000 IU

**Table 2:** Retrospective study. Demographics, routine blood biomarkers, and the serum levels of the targets in key pathways of COVID-19 cases that had no vitamin D treatments, which were separated into four groups according to serum 25OHD levels (<12 ng/mL, 12–20 ng/mL, 20–30 ng/mL, and >30 ng/mL).

		Serum 25OHD Levels				
		<12 ng/mL (L1)	12–20 ng/mL (L2)	20–30 ng/mL (L3)	>30 ng/mL (L4)	*p* Value
		*n* (%)	*n* (%)	*n* (%)	*n* (%)	
Sex	Female	31 (37.8%)	10 (24.4%)	11 (39.3%)	6 (54.5%)	0.23
	Male	51 (62.2%)	31 (75.6%)	17 (60.7%)	5 (45.5%)	
Hospital stay	<8 days	29 (35.4%)	20 (48.8%)	14 (50.0%)	6 (54.5%)	0.30
	>8 days	53 (64.6%)	21 (51.2%)	14 (50.0%)	5 (45.5%)	
ICU referral	Yes	5 (6.1%)	2 (5.0%)	1 (3.6%)	0 (0.0%)	0.82
	No	77 (93.9%)	38 (95.0%)	27 (96.4%)	11 (100%)	
		**<12 ng/mL**	**12–20 ng/mL**	**20–30 ng/mL**	**>30 ng/mL**	***p* value for MCT**
	*n*	82	41	28	11	
Age	Mean ± SD	49.70 ± 13.45	46.75 ± 11.27	54.25 ± 12.35	52.18 ± 12.01	*p* > 0.05 for all groups
Hospital stay (days)	Mean ± SD	9.40 ± 4.78	8.95 ± 4.13	8.39 ± 4.14	6.91 ± 3.36	*p* > 0.05 for all groups
Serum 25OHD levels (ng/mL)	Mean ± SD	8.16 ± 2.22	15.27 ± 2.13	23.80 ± 2.87	44.12 ± 12.87	***p* < 0.001 for all groups,** overall Post hoc power: 100%, overall Glass’ Δ: 6.84
ALT (IU/L)	Mean ± SD	32.53 ± 26.07	43.66 ± 79.13	32.45 ± 17.08	24.01 ± 15.08	*p* > 0.05 for all groups
AST (IU/L)	Mean ± SD	34.72 ± 28.79	36.67 ± 35.91	35.71 ± 18.88	27.54 ± 14.02	*p* > 0.05 for all groups
CRP (mg/L)	Mean ± SD	55.36 ± 70.44	40.85 ± 64.49	49.84 ± 53.85	25.75 ± 26.49	*p* > 0.05 for all groups
Creatinine (mg/dL)	Mean ± SD	0.84 ± 0.19	0.90 ± 0.22	0.91 ± 0.22	0.90 ± 0.25	*p* > 0.05 for all groups
Ca^2+^ (mg/dL)	Mean ± SD	8.75 ± 0.48	8.83 ± 0.53	8.89 ± 0.51	9.22 ± 0.67	**L1 vs. L4 *p* < 0.05**; *p* > 0.05 for other groups Post hoc power: 61.4%, %, Glass’ Δ: 0.98
Sodium (mmol/L)	Mean ± SD	137.76 ± 3.09	138.28 ± 3.20	136.96 ± 3.00	137.73 ± 4.47	*p* > 0.05 for all groups
Urea (mg/dL)	Mean ± SD	27.78 ± 12.46	25.67 ± 6.89	25.75 ± 8.23	26.64 ± 10.14	*p* > 0.05 for all groups
Ferritin (ng/mL)	Mean ± SD	407.55 ± 418.19	322.83 ± 304.59	455.10 ± 442.27	394.76 ± 318.01	*p* > 0.05 for all groups
Hemoglobine (g/dL)	Mean ± SD	13.48 ± 1.54	13.53 ± 1.57	13.51 ± 1.35	13.24 ± 1.23	*p* > 0.05 for all groups
Lymphocyte (×10^3^/µL)	Mean ± SD	1.61 ± 1.00	1.59 ± 0.82	1.45 ± 0.78	1.75 ± 0.93	*p* > 0.05 for all groups
Platelet (×10^3^/µL)	Mean ± SD	217.70 ± 78.02	224.95 ± 76.72	211.07 ± 54.47	210.49 ± 72.20	*p* > 0.05 for all groups
Leukocyte (×10^3^/µL)	Mean ± SD	6.94 ± 2.96	6.72 ± 3.80	5.99 ± 2.09	5.62 ± 1.75	*p* > 0.05 for all groups
D-dimer (mg/L)	Mean ± SD	2.80 ± 12.62	0.62 ± 0.55	2.49 ± 10.06	0.57 ± 0.38	*p* > 0.05 for all groups
Fibrinogen (mg/dL)	Mean ± SD	485.21 ± 178.26	426.99 ± 176.84	464.54 ± 155.50	407.79 ±167.58	*p* > 0.05 for all groups
	***n***	**18**	**18**	**16**	***n* < 3**	
PTH (pg/mL)	Mean ± SD	37.68 ± 22.87	27.10 ± 10.15	23.48 ± 11.25	-	*p* > 0.05 for all groups
Nitrate–Nitrite (µM)	Mean ± SD	12.35 ± 6.77	10.50 ± 3.89	16.11 ± 5.64	-	**L2 vs. L3 *p* < 0.05; *p* > 0.05** for other groups Post hoc power: 91.6%, Glass’ Δ: 1.44
NOS1 (ng/mL)	Mean ± SD	3.00 ± 0.85	3.73 ± 1.22	3.42 ± 1.07	-	*p* > 0.05 for all groups
DBP (ng/mL)	Mean ± SD	450.64 ± 182.61	586.10 ± 221.10	547.78 ± 174.04	-	*p* > 0.05 for all groups
IL1B (pg/mL)	Mean ± SD	6.08 ± 0.94	5.98 ± 1.44	6.34 ± 1.36	-	*p* > 0.05 for all groups
IL6 (pg/mL)	Mean ± SD	17.33 ± 33.40	14.81 ± 27.31	4.60 ± 3.33	-	*p* > 0.05 for all groups
IFNg (pg/mL)	Mean ± SD	6.08 ± 7.72	4.65 ± 4.30	3.87 ± 4.54	-	*p* > 0.05 for all groups
IL17 (pg/mL)	Mean ± SD	2.68 ± 0.57	2.56 ± 0.73	2.84 ± 0.78	-	*p* > 0.05 for all groups
LL37 (ng/mL)	Mean ± SD	19.01 ± 8.22	22.52 ± 9.49	19.33 ± 4.79	-	*p* > 0.05 for all groups
S100B (pg/mL)	Mean ± SD	6.37 ± 8.64	5.84 ± 8.94	7.86 ± 15.17	-	*p* > 0.05 for all groups
ICAM1 (ng/mL)	Mean ± SD	98.03 ± 25.50	103.89 ± 66.33	72.11 ± 23.84	-	*p* > 0.05 for all groups
VCAM1 (ng/mL)	Mean ± SD	578.17 ± 560.15	402.15 ± 302.33	370.82 ± 163.75	-	*p* > 0.05 for all groups

Bold letters indicating the group names or the significant data.

**Table 3:** Retrospective study. Demographics, routine blood biomarkers, and the serum levels of the targets in key pathways of COVID-19 cases that had no vitamin D treatments, which were separated into two groups according to serum 25OHD levels (<12 ng/mL, >12 ng/mL).

		Serum 25OHD Levels		
		<12 ng/mL	>12 ng/mL	*p* Value
		*n* (%)	*n* (%)	
Sex	Female	31 (37.8%)	27 (33.8%)	0.60
	Male	51 (62.2%)	53 (66.2%)	
Hospital stay	<8 days	28 (35%)	38 (49%)	0.08 Post hoc power: 42.9%
	>8 days	52 (65%)	40 (51%)	
ICU referral	Yes	5 (6%)	4 (5%)	0.776
	No	75 (94%)	73 (95%)	
Mortality		3 (3.7%)	1 (1.3%)	0.33
		**Serum 25OHD levels**		
		**<12 ng/mL**	**>12 ng/mL**	***p* value**
	*n*	82	79	
Age	Mean ± SD	49.71 ± 13.45	50.16 ± 12.14	0.82
Duration of hospital stay (days)	Mean ± SD	9.40 ± 4.78	8.47 ± 4.05	0.18
Serum 25OHD levels (ng/mL)	Mean ± SD	8.16 ± 2.21	22.22 ± 10.90	**<0.0001** Post hoc power: 100%, Glass’ Δ: 6.36
ALT (IU/L)	Mean ± SD	32.53 ± 26.07	36.95 ± 57.57	0.53
AST (IU/L)	Mean ± SD	34.72 ± 28.79	35.06 ± 28.34	0.94
CRP (mg/L)	Mean ± SD	55.36 ± 70.44	41.93 ± 56.86	0.19
Creatinine (mg/dL)	Mean ± SD	0.84 ± 0.19	0.90 ± 0.22	0.056 Post hoc power: 45.6%, Glass’ Δ: 0.32
Ca^2+^ (mg/dL)	Mean ± SD	8.75 ± 0.48	8.90 ± 0.55	0.057 Post hoc power: 45.3%, Glass’ Δ: 0.31
Sodium (mmol/L)	Mean ± SD	137.76 ± 3.09	137.73 ± 3.34	0.96
Urea (mg/dL)	Mean ± SD	27.78 ± 12.46	25.83 ± 7.79	0.24
Ferritin (ng/mL)	Mean ± SD	407.55 ± 418.19	384.72 ± 367.76	0.74
Hemoglobine (g/dL)	Mean ± SD	13.48 ± 1.53	13.48 ± 1.44	0.99
Lymphocyte (×10^3^/µL)	Mean ± SD	1.61 ± 1.00	1.56 ± 0.82	0.75
Platelet (×10^3^/µL)	Mean ± SD	217.70 ± 78.02	218.02 ± 68.47	0.98
Leukocyte (×10^3^/µL)	Mean ± SD	6.94 ± 2.96	6.31 ± 3.05	0.19
D-dimer (mg/L)	Mean ± SD	2.80 ± 12.62	1.31 ± 6.16	0.36
Fibrinogen (mg/dL)	Mean ± SD	485.21 ± 178.26	437.49 ± 166.76	0.12
	***n***	**18**	**34**	*p* value
PTH (pg/mL)	Mean ± SD	37.68 ± 22.87	25.40 ±10.68	**0.04** Post hoc power: 57.8%, Glass’ Δ: 0.54
Nitrate–Nitrite (µM)	Mean ± SD	12.35 ± 6.77	13.14 ± 5.51	0.65
NOS1 (ng/mL)	Mean ± SD	3.00 ± 0.85	3.59 ± 1.14	0.06 Post hoc power: 55.9%, Glass’ Δ: 0.69
DBP (ng/mL)	Mean ± SD	450.64 ± 182.61	568.07 ± 198.32	**0.04** Post hoc power: 57.2%, Glass’ Δ: 0.64
IL1B (pg/mL)	Mean ± SD	6.08 ±0.94	6.15 ± 1.39	0.85
IL6 (pg/mL)	Mean ± SD	17.33 ± 33.40	10.00 ± 20.40	0.40
IFNg (pg/mL)	Mean ± SD	6.08 ± 7.72	4.28 ± 4.36	0.37
IL17 (pg/mL)	Mean ± SD	2.68 ± 0.57	2.69 ± 0.76	0.98
LL37 (ng/mL)	Mean ± SD	19.01 ± 8.22	21.02 ± 7.71	0.39
S100B (pg/mL)	Mean ± SD	6.37 ± 8.64	6.79 ± 12.12	0.90
ICAM1 (ng/mL)	Mean ± SD	98.03 ± 25.50	88.93 ± 52.76	0.50
VCAM1 (ng/mL)	Mean ± SD	575.17 ± 560.15	386.96 ± 241.91	0.19

Bold letters indicating the group names or the significant data.

**Table 4:** Descriptive analysis of age, sex, hospital stay period and going into ICU in retrospective COVID-19 cases (without additional disease, without vitamin D treatment, and serum 25OHD <30 ng/mL) and prospective COVID-19 cases that were treated with vitamin D.

		Retrospective COVID-19 Cases (without Additional Disease, without Vitamin D Treatment, and Serum 25OHD < 30 ng/mL)	Prospective COVID-19 Cases (with Vitamin D Treatment, and Initial Serum 25OHD < 30 ng/mL)	
		*n* (%)	*n* (%)	*p* Value
Sex	Female	52 (34.4%)	80 (49.4%)	0.008
	Male	99 (65.6%)	82 (50.6%)	
Hospital stay	<8 days	63 (41.7%)	89 (54.9%)	**0.02** *
	>8 days	88 (58.3%)	73 (45.1%)	
ICU referral	Yes	8 (5.3%)	18 (11.0%)	0.07
	No	143 (94.7%)	145 (89.0%)	
Mortality		4 (2.7%)	9 (5.5%)	0.22
	*n*	151	163	
Age	Mean ± SD	50.23 ± 12.36	55.00 ± 16.45	0.004
Hospital stay (days)	Mean ± SD	8.91 ± 4.35	9.23 ± 6.54	0.30

The data was adjusted for age and sex. * The binary logistic regression analysis indicated that the significance in hospital stay (< or >8 days) did not depend on gender. Retrospective COVID-19 cases (without additional disease, without vitamin D treatment, and serum 25OHD < 30 ng/mL) had the 1.9-fold increased risk of having hospitalization longer than 8 days (*p* = 0.007, OR: 1.91, 95%CI: 1.19–3.06). Increased age was also a risk factor for hospitalization longer than 8 days (*p* = 0.023, OR: 1.03, 95% CI: 1.00–1.06). Bold letters indicating the group names or the significant data.

**Table 5:** Prospective study. Serum levels of routine blood biomarkers and key proteins of target pathways in healthy subjects, COVID-19 cases (1–3 days before vitamin D treatment) (C), COVID-19 cases in day 7 (D7), and in day 14 (D14) of vitamin D treatment.

	GROUPS				
	Healthy Subjects (H) (*n* = 23)	COVID-19 (1–3 Days before Vitamin D Treatment) (C) (*n* = 210)	COVID-19 Cases (Day 7 of vit D) (D7) (*n* = 97)	COVID-19 Cases (Day 14 of Vit D) (D14) (*n* = 95)	*p* Value for MCT (Multiple Comparison Test) Age and Sex Adjusted
Serum 25OHD levels (ng/mL) Mean ± SD	23.44 ± 9.10	16.62 ± 11.85	31.73 ± 12.29	35.46 ± 10.93	**H vs.C *p* < 0.05;** **H vs. D14 *p* < 0.001;** **C vs. D7 or D14 *p* < 0.001;** *p* > 0.05 for other groups Post hoc power: 100%
Ca^2+^ (mg/dL) Mean ± SD	8.80 ± 0.41	8.49 ± 0.87	9.06 ± 0.90	9.52 ± 0.72	*p* > 0.05 for all groups Post hoc power: 37%
PTH (pg/mL) Mean ± SD	28.97 ± 12.14	53.67 ± 114.78	49.92 ± 124.34	33.93 ± 40.15	*p* > 0.05 for all groups Post hoc power: 24%
Nitrate–Nitrite (µM) Mean ± SD	10.18 ± 6.62	16.58 ± 10.89	17.83 ± 11.67	18.53 ± 10.76	**H vs. D7or D14 *p* < 0.05;** *p* > 0.05 for other groups Post hoc power: 62%
NOS1 (ng/mL) Mean ± SD	0.81 ± 0.35	3.93 ± 2.45	3.56 ± 2.41	2.89 ± 2.00	**H vs. C *p* < 0.001;** **H vs. D7 *p* < 0.05;** **C vs. D14 *p* < 0.01;** *p* > 0.05 for other groups Post hoc power: 98%
DBP (ng/mL) Mean ± SD	258.16 ± 92.86	416.64 ± 279.55	307.67 ± 258.36	289.74 ± 270.07	**C vs. D7 or D14 *p* < 0.001;** *p* > 0.05 for other groups Post hoc power: 95%
IL1B (pg/mL) Mean ± SD	4.44 ± 0.75	7.30 ± 3.00	7.54 ± 4.19	7.07 ± 3.49	**H vs. Cor D7 *p* < 0.05;** **H vs. D14 *p* < 0.001;** **C vs. D14 *p* < 0.05;** *p* > 0.05 for other groups Post hoc power: 86%
IL6 (pg/mL) Mean ± SD	0.86 ± 0.34	19.27 ± 41.66	27.57 ± 64.32	17.82 ± 43.20	*p* > 0.05 for all groups Post hoc power: 22%
IFNg (pg/mL) Mean ± SD	1.10 ± 0.23	28.01 ± 24.63	35.66 ± 23.34	37.05 ± 21.52	**H vs. all groups *p* < 0.0001;** **C vs. D7 *p* < 0.001;** **C vs. D14 *p* < 0.0001;** *p* > 0.05 for other groups Post hoc power: 100%
IL17 (pg/mL) Mean ± SD	3.06 ± 1.03	2.09 ± 0.80	1.98 ± 1.21	2.11 ± 1.28	**H vs. all groups *p* < 0.0001;** *p* > 0.05 for other groups Post hoc power: 99%
LL37 (ng/mL) Mean ± SD	4.81 ± 2.69	18.51 ± 9.65	15.97 ± 9.23	14.76 ± 6.78	**H vs. all groups *p* < 0.0001;** **C vs. D7 *p* < 0.05;** **C vs. D14 *p* < 0.01;** *p* > 0.05 for other groups Post hoc power: 100%
S100B (pg/mL) Mean ± SD	1.43 ± 0.25	3.96 ± 6.28	3.03 ± 3.21	3.00 ± 2.56	**H vs.C *p* < 0.05;** *p* > 0.05 for other groups Post hoc power: 57%
ICAM1 (ng/mL) Mean ± SD	71.97 ± 37.92	130.48 ± 84.74	144.15 ± 77.14	145.33 ± 73.56	**H vs.D7 *p* < 0.05;** **H vs.D14 *p* < 0.01;** **C vs.D14 *p* < 0.05;** *p* > 0.05 for other groups Post hoc power: 71%
VCAM1 (ng/mL) Mean ± SD	319.84 ± 138.14	496.33 ± 354.93	571.24 ± 371.16	666.65 ± 463.34	*p* > 0.05 for all groups Post hoc power: 13%

Bold letters indicating the group names or the significant data.

**Table 6:** Prospective study. Serum levels of routine biomarkers in COVID-19 cases without vitamin D treatment (C), COVID-19 cases in day 7 (D7), and in day 14 (D14) of vitamin D treatment.

	COVID-19 Cases (1–3 Days before Vitamin D Treatment) (C) (*n* = 209)	COVID-19 Cases (Day 7 of Vit D) (D7) (*n* = 99)	COVID-19 Cases (Day 14 of Vit D) (D14) (*n* = 86)	*p* Value for MCT
ALT (IU/L) Mean ± SD	29.08 ± 21.42	49.23 ± 44.76	53.22 ± 62.64	**C vs. D7 or D14 *p* < 0.001**; *p* > 0.05 for other groups
AST (IU/L) Mean ± SD	31.44 ± 23.41	35.61 ± 26.62	31.68 ± 29.86	*p* > 0.05 for all groups
CRP (mg/L) Mean ± SD	50.68 ± 66.41	28.13 ± 49.08	10.96 ± 27.27	**C vs. D7 or D14 *p* < 0.001;** **D7 vs. D14 *p* < 0.001;** *p* > 0.05 for other groups
Creatinine (mg/dL) Mean ± SD	1.03 ± 0.65	1.08 ± 1.02	0.87 ± 0.27	*p* > 0.05 for all groups
Sodium (mmol/L) Mean ± SD	137.08 ± 8.51	139.28 ± 3.69	139.63 ± 3.24	**C vs. D7 or D14 *p* < 0.001;** *p* > 0.05 for other groups
Urea (mg/dL) Mean ± SD	35.46 ± 22.64	40.77 ± 28.98	32.22 ± 16.44	*p* > 0.05 for all groups
Ferritin (ng/mL) Mean ± SD	408.15 ± 474.26	421.19 ± 498.75	252.52 ± 299.45	*p* > 0.05 for all groups
Hemoglobi Mean ± SD	12.43 ± 1.89	12.27 ± 1.80	12.69 ± 1.75	*p* > 0.05 for all groups
Lymphocyte (×10^3^/µL) Mean ± SD	1.56 ± 0.82	1.60 ± 0.86	1.84 ± 0.65	**C vs. D14 *p* < 0.001;** **D7 vs. D14 *p* < 0.05;** *p* > 0.05 for other groups
Platelet (×10^3^/µL) Mean ± SD	210.80 ± 81.10	296.25 ± 124.71	296.67 ± 91.07	**C vs. D7 or D14 *p* < 0.001;** *p* > 0.05 for other groups
Leukocyte (×10^3^/µL) Mean ± SD	7.51 ± 7.55	8.31 ± 6.63	7.60 ± 3.02	**C vs. D14 *p* < 0.01;** *p* > 0.05 for other groups
D-dimer (mg/L) Mean ± SD	0.99 ± 1.21	1.08 ± 1.22	0.76 ± 0.83	*p* > 0.05 for all groups
Fibrinogen (mg/dL) Mean ± SD	469.60 ± 172.43	449.55 ± 148.01	375.42 ± 116.03	**C vs. D14 *p* < 0.001;** **D7 vs. D14 *p* < 0.001**; *p* > 0.05 for other groups

Bold letters indicating the group names or the significant data.

**Table 7:** Prospective study. Serum levels of biomarkers of vit D metabolism and inflammation in healthy subjects, COVID-19 cases (1–3 days before vitamin D treatment), COVID-19 cases in day 7 and in day 14 of vitamin D treatment.

Groups	Ca2+	PTH	Nitrate–Nitrite	NOS1	DBP	IL1B	IL6	IFNg	IL17	LL37	S100B	ICAM1	VCAM1
Healthy subjects (*n* = 23)	NC	*p* = 0.08 95% CI: −0.68 to 0.05, r^2^ = 0.14	NC	NC	NC	NC	NC	NC	NC	NC	NC	NC	NC
COVID-19 cases (1–3 days before vitamin D treatment) (*n* = 210)	*p* = 0.049 95% CI: 0.0006 to 0.27, r^2^ = 0.02	*p* = 0.02 95% CI: −0.29 to −0.03, r^2^ = 0.03	*p* = 0.047 95% CI: −0.27 to −0.002, r^2^ = 0.02	*p* = 0.06 95% CI: −0.26 to 0.005, r^2^ = 0.02	*p* = 0.03 95% CI: 0.01 to 0.28, r^2^ = 0.02	*p* < 0.0001 95% CI: 0.15 to 0.40, r^2^ = 0.08	NC	*p* = 0.06695 95% CI: −0.026 to 0.0009, r^2^ = 0.016	NC	NC	NC	*p* = 0.0003 95% CI: −0.37 to −0.12, r^2^ = 0.06	NC
COVID-19 cases (day 7 of vit D treatment) (*n* = 97)	NC	*p* = 0.074 95% CI: −0.37 to 0.02, r^2^ = 0.03	NC	*p* = 0.043 95% CI: 0.007 to 0.39, r^2^ = 0.04	*p* = 0.043 95% CI: 0.007 to 0.39, r^2^ = 0.04	NC	NC	NC	NC	*p* = 0.005 95% CI: 0.09 to 0.46, r^2^ = 0.08	NC	NC	NC
COVID-19 cases (day 14 of vit D treatment) (*n* = 95)	NC	NC	NC	*p* = 0.023 95% CI: 0.03 to 0.42, r^2^ = 0.05	*p* = 0.033 95% CI: 0.02 to 0.41, r^2^ = 0.05	NC	NC	NC	NC	*p* = 0.008 95% CI: 0.07 to 0.45, r^2^ = 0.07	NC	NC	NC

NC: No correlation.

## Data Availability

All data generated or analyzed during this study are included in this published article.
